# Exploring EEG spectral and temporal dynamics underlying a hand grasp movement

**DOI:** 10.1371/journal.pone.0270366

**Published:** 2022-06-23

**Authors:** Sandeep Bodda, Shyam Diwakar

**Affiliations:** 1 Amrita Mind Brain Center, Amrita Vishwa Vidyapeetham, Kollam, Kerala, India; 2 Department of Electronics and Communication Engineering, School of Engineering, Amrita Vishwa Vidyapeetham, Kollam, Kerala, India; Georgia State University, UNITED STATES

## Abstract

For brain-computer interfaces, resolving the differences between pre-movement and movement requires decoding neural ensemble activity in the motor cortex’s functional regions and behavioural patterns. Here, we explored the underlying neural activity and mechanisms concerning a grasped motor task by recording electroencephalography (EEG) signals during the execution of hand movements in healthy subjects. The grasped movement included different tasks; reaching the target, grasping the target, lifting the object upwards, and moving the object in the left or right directions. 163 trials of EEG data were acquired from 30 healthy participants who performed the grasped movement tasks. Rhythmic EEG activity was analysed during the premovement (alert task) condition and compared against grasped movement tasks while the arm was moved towards the left or right directions. The short positive to negative deflection that initiated around -0.5ms as a wave before the onset of movement cue can be used as a potential biomarker to differentiate movement initiation and movement. A rebound increment of 14% of beta oscillations and 26% gamma oscillations in the central regions was observed and could be used to distinguish pre-movement and grasped movement tasks. Comparing movement initiation to grasp showed a decrease of 10% in beta oscillations and 13% in gamma oscillations, and there was a rebound increment 4% beta and 3% gamma from grasp to grasped movement. We also investigated the combination MRCPs and spectral estimates of α, β, and γ oscillations as features for machine learning classifiers that could categorize movement conditions. Support vector machines with 3^rd^ order polynomial kernel yielded 70% accuracy. Pruning the ranked features to 5 leaf nodes reduced the error rate by 16%. For decoding grasped movement and in the context of BCI applications, this study identifies potential biomarkers, including the spatio-temporal characteristics of MRCPs, spectral information, and choice of classifiers for optimally distinguishing initiation and grasped movement.

## 1. Introduction

Patients with amyotrophic lateral sclerosis (ALS) or spinal cord injury (SCI) are reported to have significant loss of voluntary motor control and extensive dysfunction of upper and lower limbs [1–4]. Rehabilitation using robotic or functional electrical stimulation could facilitate novel therapeutic strategies for such conditions. Integration of signals could also be a potential goal for Brain-Computer Interfaces (BCI), allowing the transfer of control information from a prosthesis onto the brain tissue [5–7].

Neural correlates of voluntary grasped movement are relevant in the development of modern prosthetics and towards Brain-Computer Interface (BCI) research [8–10]. Insights from electroencephalography (EEG) recordings and their underlying neurophysiological processes involved in motor tasks can help decode movement and its functional interpretation. EEG signal components related to movement allow noninvasive measurements, making them suitable for natural BMIs [5, 6]. In addition, these correlates are measurable in paralyzed patients [3, 4, 7, 11]. For example, the assessment of grip forces [12], movement direction, and lifting paradigms [13] have allowed to objectively and quantitatively detect deficits in patients with Multiple Sclerosis (MS). Studies [14–16] have also focused on low-frequency EEG components to understand how movement trajectories [17] and grasp types [18] were encoded.

EEG-based studies have explored movement initiation or intention and execution to be associated with μ/α and β rhythms [19], although the role of higher frequency oscillations in movement execution remains to be critically explored. The modulation of β rhythms and oscillations in the context of sensorimotor state maintenance and motor function has been previously reported [20, 21]. However, there is little information related to cortical zone-dependent microstates and their movement-related cortical potential (MRCP) components and their variability during resting states for reach, grasp, and grasped movement tasks performed using a human arm. Readiness potentials, and time delays to occurrences of motor-related events while simultaneously comparing motor imagery and motor execution spectral components in EEG can extend current knowledge for developing BCIs [10]. Event-related (de)synchronisation ERD/S and movement-related cortical potentials (MRCPs) have been employed to assess and detect movement intention, execution and imagery in several studies [22–26]. Among the neural correlates of voluntary movement intention, studies had reported the role of a component of MRCP, the slow negative potential [27, 28]. MRCPs have been observed as low-frequency components within EEG signals, computed by averaging several trials commencing 2s before voluntary movement [29, 30]. The readiness potential (RP) [31], an MRCP component, was perceived with a larger amplitude at contralateral central regions around 400ms before the movement onset. In the case of conscious voluntary movement, readiness potential was known to precede 500-800ms before the onset of movement [32, 33]. MRCPs serve as reference attributed to cortical excitability [34–36] occurring before the movement. Also, MRCPs have been used as biomarkers of movement intention and movement tasks in ALS [37], stroke [25], and Parkinson’s disease [38, 39] patients.

The neural components correlated to hand-reaching that occurred within the medial parieto-frontal circuit including the medial intraparietal area (mIP) at the boundaries with area V6A and the dorsal pre-motor areas have been explored [40]. Neural activity related to grasping was reported to occur in the lateral parieto-frontal circuit involving the anterior intraparietal area (AIP) primarily and the dorsal (PMd) and the ventral (PMv) regions of pre-motor areas [41–43]. A significant role was known to be identified in the human AIP (hAIP) during grasping tasks [44–49]. It was demonstrated [50] that grasp-related neural activity was observed in the ventral premotor F5, parietal AIP, the cortical F1 areas pertaining to the hand field and in the several regions of the somatosensory cortex. An intrinsic spatial optical imaging (ISOI) study [51] had explored spatial domains that were active in M1 and S1 in response to reaching and grasping in macaque monkeys.

Assessing synchrony and desynchrony of rhythmic oscillations had allowed dissecting cortical preparation or execution of voluntary movements [52–54]. In the motor cortex, μ rhythms in the α band [22, 55], and attenuation of the α-band were observed during the preparation or execution of voluntary movements, which was also accompanied by a decrease in the β band decrease, known as event-related desynchronization (ERD). Further, a rebound of the β-band after the movement, known as event-related synchronization (ERS) [22] was used to detect changes in movement execution. From an application standpoint, ERD/ERS features allow the reuse EEG signal information within the context of a brain-machine interface (BMI) and similar methods in the anthropometric articulation of external devices [53, 54, 56–58]. Control paradigms were employed to operate wheelchairs using decoded ERD/ERS [59, 60] information. During the movement preparation and execution, ERD was explored for investigating cortical modifications and was shown the delayed ERD in Parkinson’s Disease (PD) patients compared with healthy subjects over the contralateral sensorimotor areas [22, 61]. In the S1 area, for self-paced finger or wrist-extension movement, a decrease in 10–14 Hz frequencies and a transient increase in activity (ERS) after the movement for 20 and 90 Hz frequencies were observed [62]. An increase in the 40 Hz gamma-band activity (ERS) over the central scalp region was reported during both simple response time (SRT) and complex response time (CRT) task reactions [63, 64]. Studies show correlations in the slow (6–11 Hz) and to the higher © band region of (60–120 Hz) [65, 66] corresponding to any of the 4 fingers being moved and their flexion and trajectories of finger movements during grasping.

Other than μ or β band activity, studies have reported γ band activity in the motor and premotor areas from intracranial electrode recordings and electrocorticogram recordings [67–69]. The subdural recordings of electrocorticography (ECoG) signals performing visuomotor tasks designed to activate representations of different body parts had shown © bands were distinct in the sensorimotor cortex and the ERS of γthe band has been observed during or just before the motor response and lasted for a short time and ended before the completion of the motor response. The temporal differences associated with the γ bands had implicated functional associations with motor performance [64]. During the execution phases of movement, γ band synchronisation was also reported, and γ clusters contralateral to movement have been observed [70, 71]. The findings [70] of Ramos-Murguialday and colleagues had indicated that active movement could be decoded using a low γ-band in EEG after filtering EMG artifacts from channel data.

There has been an increased demand for wearable technologies and especially, limited-channel systems for personal use. Comparing clinically graded systems and consumer systems and the applications [72–74] have been reported. The application of these consumer systems in research has not been extensively explored. It was also crucial to explore whether a limited-channel acquisition could provide interpretable data to assess the neural signatures while exploring grasped movement.

In other BCI studies [75–80], various grasp movements have been used in actual and imagined motor tasks but due to task complexity, they have not been interpreted for BCI and related neurophysiological studies. The MRCPs and spectral oscillations relationship have not been investigated in detail and remain uncertain, although both phenomena accompany sensorimotor activity, and their underlying neural circuits have been hypothesized to reside within the same anatomical structures. A few studies have explored their relationship from a neurophysiology perspective [81–84]. Through this study, the neural correlates could provide complementary information on associated grasped movement tasks where MRCPs could be related to cortical excitability shifts and ERD/ERS to the gating of task-specific thalamocortical circuits [85]. In the context of early movement prediction and detection, the number of studies addressing the relation of MRCP and ERD/ERS morphology and the classification of such data are limited [2, 24, 25].

Since lower resolution EEG was less expensive and easier to implement than the other temporally precise methods, and with the limits of EEG spatial resolution not well understood, whether specific EEG electrodes are sufficient for movement-related spatial information remains unanswered. This study explores the EEG dynamics by employing a novel protocol to understand movement intention and execution during grasped movement tasks performed in the left and right directions. While exploring the activity space underlying grasping controlled by parieto-frontal circuits [86] and central regions, this paper also attempts to relate the variations in movement initiation and grasped movement by developing classification models based on features of temporal and spectral correlations specifically with a combination of α, β, and lower γ frequency bands and readiness potentials for grasped-movement.

In our study, the rebound activity of β and γ oscillations after the movement and the slow negative deflection amplitude shift in the temporal domain were explored as these features could be potential cortical activity biomarkers for grasped movement. In this paper, we also evaluated feature-based classification of pre-movement and grasped movement using machine learning and have enumerated ranked features from these tasks that may be relevant for constructing efficient classifiers for clinically relevant movement EEG data.

## 2. Methods

Thirty healthy volunteers aged 18 to 30 years (mean age = 22.32 ± 1.92 years) participated in this study. This non-invasive study was reviewed and approved by the institutional ethics committee at the university. All subjects involved in this study were without any known prior medical conditions and with normal or corrected-to-normal vision. All subjects were explained the aim of the study and signed informed consent were collected before their participations in the recordings. The tasks included were grasped movement of bottle towards using left hand towards the left direction and with right-hand towards the right direction.

### 2.1 Experimental paradigm

**Fig 1:**
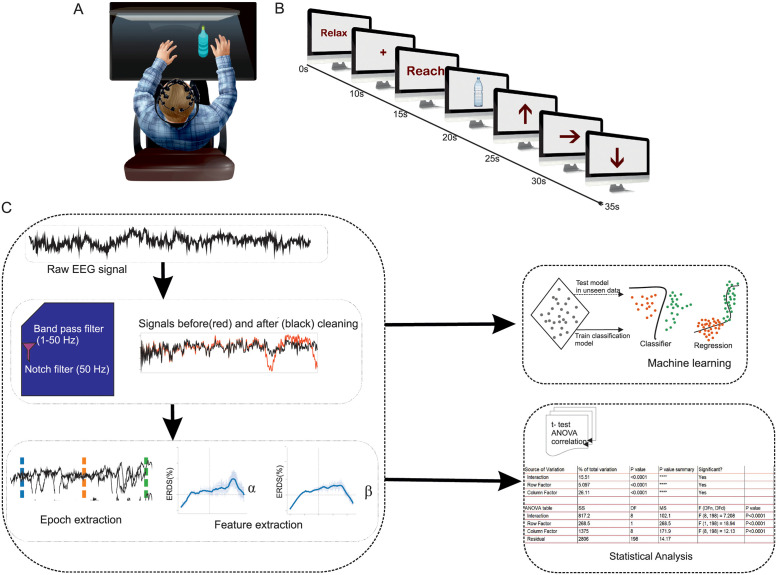
Schematic representation of experimental protocol and configuration. (A) Sitting posture of the subject during the neurophysiological recording (B) The timeline points to the temporal reference for various tasks initiated with a ‘relax’ state that started at 0s, “+” visual cue at 10s for the subject to be ‘alert’, at 15s ‘ Reach’ cue indicated the subject to reach for the object placed in the front of the subject, and at 20s ‘Object’ cue appeared to indicate the subject to grasp the object, followed by “arrow” cue at 25s to lift the bottle, consequently followed by another ‘arrow’ cue at 30s that suggested the direction in which the object was to be moved and a ‘down arrow’ cue at 35s that suggested to place the grasped object back at rest. (C) EEG Data processing analysis workflow involved the filtering of raw signal, epoch extraction, feature extraction and building a machine learning model based on processed features.

The experimental setup and the task paradigm were adapted from [16] (see Fig 1). A cue-based paradigm (Fig 1B) was employed where subjects were presented with visual cues using a slide-based presentation for 45 seconds. During the recordings, the subjects were seated in a comfortable chair and the computer display was at eye level and an object to grasp was placed at a random distance of 69–82 cm (approximately the length of reaching the object by the subject’s arm) from the subject (Fig 1A). Subjects were asked to place their left or right arm parallel to the table while resting on the hand-rest of the chair. The object, a 6.5 cm diameter rigid bottle was positioned on the centre of the table.

Prior to the experiment, participants were provided training to adapt to the task by progressive steps of hand movement by grasping the object. Before recordings, the subjects repeated the same procedure three or four times until they attained familiarity to perform the task.

The experimental paradigm was conducted as outlined in the following steps.

1. All trials commenced with a relaxation phase (blank screen), considered as a reference or baseline signal for the analysis
2. The subject was asked to relax for ten seconds.
3. The subject was alerted for the following task by a ‘+’ sign cue for 5 seconds.
4. The word ‘Reach’ as a cue was shown for 5 seconds, indicating the subject to reach the object.
5. Then an image of a bottle was shown to the subject, indicating to grasp the bottle placed in front of the table. This cue was presented for five seconds
6. An upward arrow cue (↑) was shown for next 5 seconds indicating to the subject to lift the bottle to the chin level.
7. This was followed by a leftwards arrow cue (←) for five seconds; subject was then instructed to move the bottle towards left direction using the left hand.
8. Following this, a down-wards arrow cue (↓) was shown for 5 seconds indicating to the subject to place the object back down on the table
9. A blank screen was shown as an indication of the end of the experiment trial

The same steps were repeated for right-hand right direction movement task as well.

The tasks were carried out using both hands for the two movement directions in trials defined as right-hand leftward direction (RHLD), right-hand rightward direction (RHRD), and left-hand leftward direction (LHLD), and left-hand rightward direction (LHRD). However, only LHLD and RHRD have been considered for the analysis.

### 2.2 EEG data acquisition

The study included the acquisition of EEG data by placing EEG sensors on the subject’s scalp. For clinically relevant data, we used a 32-electrode commercially available device (Neuroelectrics, Barcelona, Spain) positioned on the scalp according to the 10–20 international system with a sampling rate of 500Hz.

Considering commercial and limited electrode platforms, we also employed a 14-electrode device (EMOTIV EPOC+) with a sampling rate of 128 Hz. EEG signals were recorded for 10 subjects using a 32- electrode device. For each subject, four trials were performed per task. So, a total of 40 trials were performed for 10 subjects. Due to the loss of data packets, EEG data of 12 trials were discarded. Using the 14-electrode device, EEG signals were recorded for 20 subjects. In this, 10 trials were performed for 6 subjects, 5 trials for 12 subjects, 13 trials for 1 subject, and 2 trials for 1 subject have been carried out and EEG signals were recorded. Out of 175 trials, 163 were used for the data analysis, and 12 trials were rejected due to the loss of signal packets during the recording.

### 2.3 Offline processing of EEG data

Data analysis was performed using custom scripts written in Python incorporating the ‘mne’ package functions [87]. To obtain the spectral components initially the data was high pass filtered (1Hz) to minimise the drifts [88] and the reference (mean) was subtracted, further the data was detrended [89] and bandpass filtered using an FIR filter [90, 91] of order 20 within the range, 1Hz—60 Hz and notch filter was applied to remove line noise in the range of 50 Hz. Independent Component Analysis (ICA) [92, 93] was used to eliminate the EOG, EMG artifacts from the data. ICA considered n number of linear mixtures *X*_*1*_, *X*_*2*_……., *Xn*, n number of components in this case total number of channels have considered n = 32 number of components. signal X:

X=As (1)

*A* represents mixing matrix with size n×n and s was the vector of independent components. The mixing effect, after computing the matrix generated the independent components

y=wX≅S (2)

To obtain the independent components in this study, we used the infomax algorithm based on general optimisation principle for neural networks and other processing systems [93, 94]. The algorithm determined weights based on the maximation of output entropy of a neural network with nonlinear outputs:

$$w_{k+1}=w_{k}+\mu_{k}\left[I-2g\left(y_{k}\right)y_{k}^{T}\right]w_{k}$$ (3)

where: y–matrix of source estimation (y = Wx); k–number of iterations; I–the identity matrix; μ_*k*_−learning rate which depended on k; g (.)–a nonlinear function. *g(y)* logistic function.


$$g\left(y\right)=\frac{1}{1+\;e^{-y}}$$ (4)

Further, data was segmented into non-overlapping epochs of 2s for the timelines of 0s - 10s (Relax Task), 10s-15s (premovement task), and 30s -35s (Left hand left direction movement / Right hand right direction movement). Consequently, the segmented data was used to estimate the spectral features. The spectral estimations of each rhythm were quantified using multitaper power spectral density (PSD) estimation [95, 96]. The standard multitaper PSD [97] consisted of a series of steps: multiplying each data segment by each taper, applying Fourier analysis to these products, averaging over the tapers within each segment, and averaging over the segments. The PSD was estimated for the frequency ranges of δ, θ, α/μ, β, and γ bands. The global field power (standard deviation) across the regions (central, frontal and parietal) of electrodes was obtained.

For temporal cortical potential components [29], The MRCPs typically occur at frequencies of around 0–5 Hz [98]. To minimise the drifts from the raw data reference (mean) was subtracted, further the data was detrended [89] and bandpass filtered using an FIR filter [90, 91] of order 20 within the range of 0.1Hz- 5 Hz. Further, Multiple recordings of the same trials must be taken and then averaged across the trials for extraction of MRCP from EEG traces. By averaging, the background noise is cancelled out leaving only the MRCPs, when the data from multiple trials is filtered to eliminate the higher frequency activity.

### 2.4 Statistical measures

Correlation analysis was carried out to determine the best electrode positions for the analysis of MRCPs. The data from channels (C3, C4, F3, F4, P3, P4) were selected and used for correlation analysis. The relation for electrode positions in different regions of cortical regions from MRCPs was analysed using Pearson correlation coefficient. For each electrode channel and N scalar observations (time points), the correlation coefficient was computed.

$$\rho \left(x,y\right)=\frac{1}{\left(N-1\right)}\sum_{i=1}^{N}\left(\frac{\bar{x_{i}-\mu_{x}}}{\sigma_{x}}\right)\left(\frac{\bar{y_{i}-\mu_{y}}}{\sigma_{y}}\right)$$ (5)

Where x and y were two variables (electrode channels) corresponding to two electrode positions under analysis. The μ_x_ and σ_x_ values were mean and standard deviation of x (channel 1) and the μ_y_ and σ_y_ were the mean and standard deviation of y (channel 2) respectively. The goal of the correlation analysis was to identify the optimal electrode position for the relax, premovement and movement tasks. Further, the correlated channels were compared to test the significance of correlated channels for each task using multiple t-test statistics. Statistical significance was determined using the Holm-Sidak method, with alpha = 0.05. Each row (correlated pair combination of electrode) was analysed individually, without assuming a consistent SD.

The association between frequency band oscillations in the three tasks and the subject’s gender was tested using χ^2^ analysis. The value of χ^2^ was estimated using the formula,

$$\chi^{2}=\frac{({F_{O}-F_{E})}^{2}}{F_{E}}$$ (6)

where F_O_ was the observed frequency count and F_E_ was expected frequency count of dependent features, the confidence interval chosen was 0.05. Similar analysis was performed to find the association between frequency band and tasks (the average count of the frequency bands was used in the contingency table for analysis).

To better understand the relationship between changes in PSD for central electrode regions across different frequency bands for all 28 trials, PSD for tasks (relax, premovement, movement) was compared. A two-way repeated measure of ANOVA with Tukey’s multiple comparison posthoc test was performed on the average of each feature extracted from the relax, pre-movement and grasped movement conditions. Similarly, we compared the frequency band features for the task’s male and female subjects for all the trials, to identify the statistical significance of the features discriminating among the male and female subjects for each task using two-way ANOVA analysis. All the statistical analysis was performed using GraphPad Prism [99].

### 2.5 Machine learning

Using Decision trees (DT) [100–102], support vector machines [103, 104] with different kernels, and multilayer perceptron [105, 106] with different activation functions and solvers, classification was performed on the feature-combined dataset including both spectral and temporal (RPs) information for the premovement vs movement tasks and left-hand grasped-movement vs right hand grasped movement tasks. Here, DT (criterion: ‘*Gini*’, splitter: ‘*best’*), SVM model [107–109] (regularisation parameter C = 1.0, Kernel: linear, polynomial and radial basis function, degree = 3) and for MLP (activation function: logistic sigmoid function, quasi-Newton method as a solver: ‘lbfgs’, hidden_layer_sizes: (35,2), maximum number of iterations = 300). The implementation of the algorithms was based on the scikit-learn [110] python package and was chosen for the classification of grasped movement data.

Two EEG datasets consisting of 270 samples (135 samples premovement, 135 movement trials) and 56 samples (trials or instances) (28 premovement, 28 movement trials) respectively were used in this machine learning analysis. The two datasets contained 67 and 74 features as columns which were pre-processed values from the raw data. The classifier models were trained, and accuracy was estimated using 10-fold cross-validation and the datasets had split into training and testing samples with a random choice. To explore the differences in the accuracies of the datasets, Wilcoxon signed ranked t-test [111] was employed, and the t-test had indicated that there were no significant effects (p = 0.65) in the classification accuracies when using electrodes from two different headsets data (also see S4 Fig in S1 File).

#### 2.5.1. Pre-classification feature selection for pre-movement and grasped movement tasks

**Fig 2:**
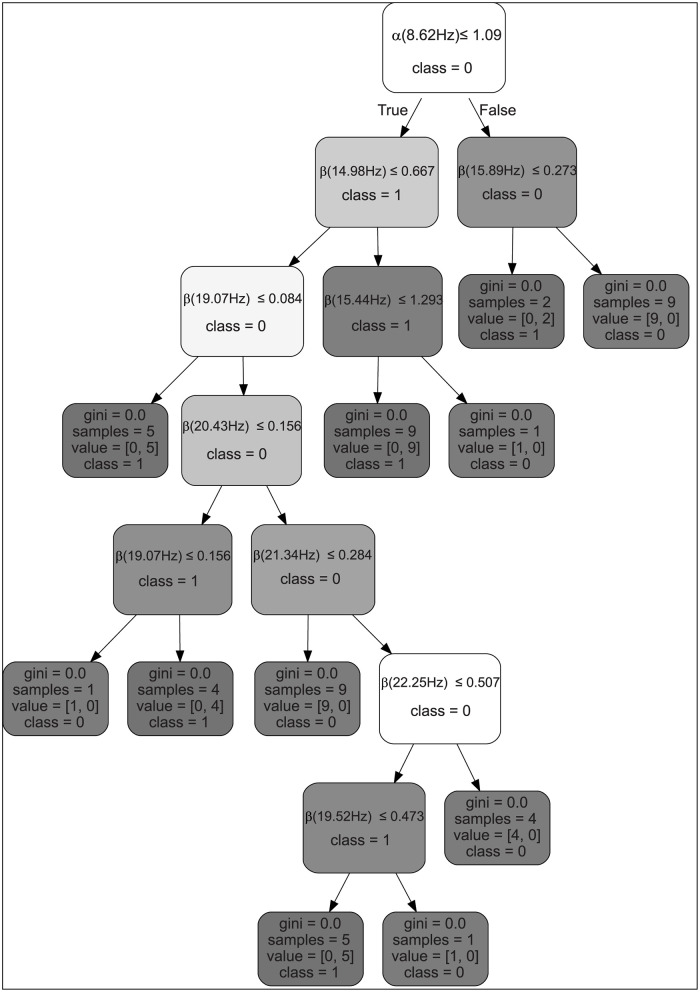
Feature ranking of surface EEG signals from central electrodes C3 and C4 showed accuracy depended on α sub-band 8.62 Hz and β sub-bands 14, 15, 19, 20, 21, 22 Hz while classifying movement (gray) against premovement (light grey/white) class labels (Class 0 is premovement, Class 1 is grasped movement).

20 best-ranked features in the dataset were identified using the feature selection ranker search algorithm [112, 113], which indicated that the frequency sub-bands of μ/α (7–10 Hz), β (18, 25–29 Hz), γ (30–32 Hz) and related potential (see Fig 2).

## 3. Results

### 3.1 MRCP-related time-domain variations allow to decode grasped- movement

**Fig 3:**
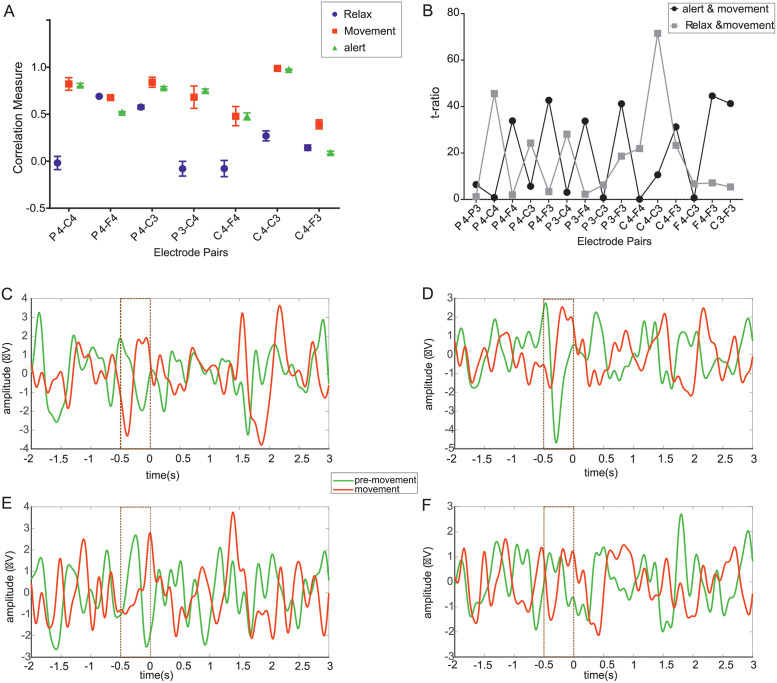
Temporal correlations across MRCPs from EEG electrode regions differentiate movement tasks. (A) Correlation measures for central regions (C3-C4) electrode pair has indicated higher correlation (r = 1) for movement and ‘alert’ condition tasks whereas for a lower correlation measure (r) of 0.3 was estimated for the ‘relax’ task., P4-F4 electrode pair has indicated a measure of 0.5 for premovement and relax tasks and 0.6 for movement task. The electrode combination of C3-C4 for movement and “alert” condition had higher correlation measure compared to P4-C4, P4-F4, P4-C3, C4-F4, C4-F3 combinations. (Blue circle–“relax task”, Red square–“movement task”, Green triangle–“alert/premovement” task (B)Correlation measure of various electrode pair combinations was statistically compared for the tasks using t-test. and C3-C4 electrode combination were significantly different for “relax” and movement tasks indicating central region electrodes could be decisive in differentiating “relax” and grasped movement tasks. (C) potential amplitude decreases in 296% from premovement to movement in the central electrode regions (D) Parietal regions has shown 164% decreased in amplitude (E) Frontal regions has shown 133% decrease and (F) Occipital regions has shown 230% decrease in potential amplitude before 500ms to onset of movement. (Green line–“premovement task” and Red Line indicates “movement” task).

Associated to C3-C4 electrodes (see S1 Fig in S1 File), the postcentral, precentral gyrus and the primary motor cortex (M1) has a role in the initiation and fine control of movement; and, in this study, the central electrode regions (C4-C3) reported a higher correlation measure (r = 1) for movement and “alert”/pre-movement conditions compared to “relax” or no-movement state (r = 0.3) (Fig 3A, also see S4 Table in S1 File for other electrode regions). A combination of parieto-central and frontocentral region electrodes showed a correlation measure of 0.8 and 0.4 for movement and premovement conditions respectively (Fig 3A). Parieto-frontal (P4-F4) regions reported a correlation measure of 0.5 for the premovement/ “alert” and “relax” tasks, 0.6 for the “movement” task. The correlation-based analysis recommended C3-C4 combination for premovement and movement tasks due to the higher values compared to P4-C4, P4-F4, P4-C3, C4-F4, C4-F3 combinations.

A t-statistic value of 70 for the “relax” and the movement tasks at the central electrode regions indicated that the two tasks were significantly different from each other for C3-C4 (Fig 3B). P4-C4 combination of electrode regions reported a t-statistic of 45 for “relax” and “movement” tasks and P3-F4 and P4-F4 regions indicated a t-statistic value of less than 10. F4-C3, F4-F3, and C3-F3-central regions showed t-statistic values of less than 10 suggesting the tasks may not be different from each other across these electrode pairs. Among the central (Fig 3C), parietal (Fig 3D), frontal (Fig 3E), and occipital regions (Fig 3F), although no change in amplitude of the MRCP was observed during “alert” or “premovement” condition, a shift from the negative to positive wave was observed for movement tasks before movement (-0.5s) until the onset of movement (Fig 3).

### 3.2 Increased motor cortical β and γ frequency bands act as biomarkers for detecting movement during a grasped movement task

Scalp topographies indicated the presence of the attenuated α band (ERD) and amplified β band (ERS) modulations during movement initiation in the central parietal regions over the different sub-band regions (see S1 Fig in S1 File). 15–25 Hz β sub-band regions showed activation along the precentral gyrus, postcentral gyrus (corresponding to Central C3, C4 electrode locations) (See S1 Table in S1 File for more electrode locations), and superior lateral occipital cortex (corresponding to parietal P3, P4 electrode locations) (see S1A and S1B Fig in S1 File).

#### 3.2.1. Characterisation of μ/α oscillations

**Fig 4:**
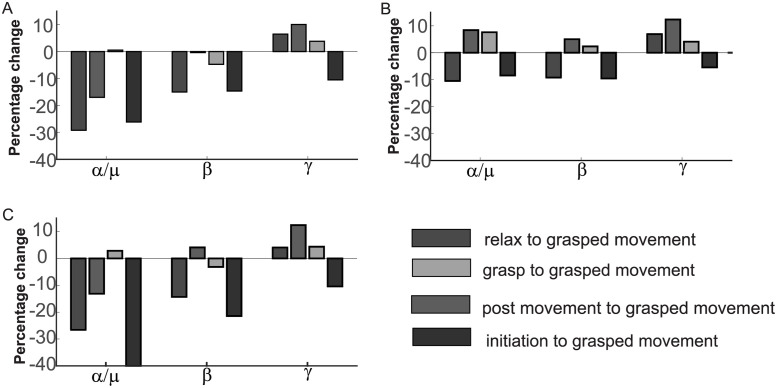
Increased post movement activity by rebound β and γ oscillations. (A) α/μ oscillations show 26% decrease from initiation to grasp movement 29% decrease from relax to grasp movement and 16% decrease from grasp movement to post movement in the central electrode regions. 14% and 10% decrease in beta and gamma oscillations from initiation to grasp movement and rebound increment of 14% beta and 20% gamma for post grasp movement. (B) Frontal region has shown 7% increase of alpha oscillations from grasp to grasped movement and 8% increase post movement (C)Parietal region electrodes has shown 21% and 20% decrease from initiation to grasp movement and rebound increment of 4% and 12% post movement. (For inter subject variability see S3 Fig in S1 File).

A 26% decrease in the μ/α oscillations was estimated for initiation to grasp movement, a 16% decrease was observed during grasp movement to post-movement and a 29% decrease was prominent during “relax” to grasp movement tasks across central electrode regions (Fig 4A). Compared to the central regions (corresponding to C3-C4 positions), frontal regions (corresponding to F3-F4 positions) reported a 7% increase in μ/α oscillations for grasp to grasped movement, and an 8% increase post-movement (Fig 4B).

#### 3.2.2. Characterisation of β and γ

There was a decrease of 14% of β and 10% of γ oscillations during movement initiation to grasped movement, and a rebound increment of 14% of β and 20% of γ frequencies post-movement were observed (Fig 4A). 9% decrease of β and 5% decrease of γ rhythms from initiation to grasped movement and rebound increment 4% and 12% for β and γ oscillations were observed in the frontal regions. In the parietal regions, 21% for β and 10% for γ decreased and rebound increment of 4% and 12% for β and γ oscillations. (Fig 4C). Also, a 2% increase for grasp to grasped movement and a 13% decrease post-movement for μ rhythms (Fig 4C) were observed.

A 7% increase in θ oscillations was observed post-movement in the middle frontal gyrus and frontal pole (corresponding to frontal lobes). During the task change from grasp to grasped movement, theta oscillations decreased by 20% in the central region (precentral gyrus and postcentral gyrus) and 30% in the parietal (superior Lateral Occipital Cortex) regions (See S2 Fig in S1 File).

The three tasks with the oscillations (α, β, and γ) were compared using the χ^2^- test. The χ^2^ value = 21.51 (degree of freedom = 4), indicated the percentage of brain oscillations varied among premovement, left movement, and right movement tasks rejecting the presumed null hypothesis and were statistically significant (p = 0.003). Among genders, with χ^2^ statistic as 89.43 (degree of freedom = 8), p <0.0001, the data showed significant variations among male and female subjects (see S2 Table in S1 File).

**Fig 5:**
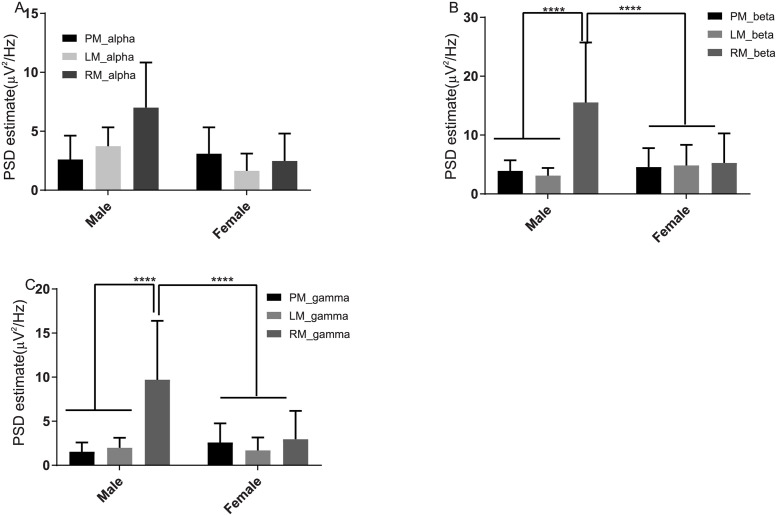
Significant left and right movement in male and female subjects in the central region electrodes among β *&* γ oscillations. (A, B, C) Variability between the male and female subjects for the tasks of premovement (PM) left movement (LM) and right movement (RM). (A) α oscillations for premovement task of male subjects (2.609 ± 2.025) female subjects (3.091 ± 2.245) have not shown any statistical significance similarly for left movement and right movement (p value >0.9999), (B) β and (C) γ oscillations have shown significant differences were presented with p value < 0.0001 (12 sample trials of Male subjects, 12 sample trials of female subjects).

The Tukey test (see S3 Table in S1 File) among male and female subjects indicated that β oscillations of right-hand grasped movement from male participants were significantly different from β oscillations of premovement, left hand grasped movement, and right-hand grasped movement from female participants (p-value <0.0001) (See Fig 5).

### 3.3 Machine learning and task-specific classification of grasped movement

Classification for discriminating tasks on the EEG datasets from the 14-channel electrode and 32 channel electrodes across 170 samples and model evaluation was performed. SVM (polynomial order 3) gave the best accuracy in most classification tasks, with the best mean accuracy of 60% compared to SVM (with radial basis function), multi-layer perceptron (MLP), and decision tree (DT) algorithms (See S4A Fig in S1 File). With the test dataset of 100 samples, the SVM polynomial kernel had a higher performance rate of 54% compared to other algorithms (see S4B Fig in S1 File). When comparing left movement and right movement, only 49% training accuracy was noted with SVM (see S5 Table in S1 File for accuracy rate with other algorithms).

**Fig 6:**
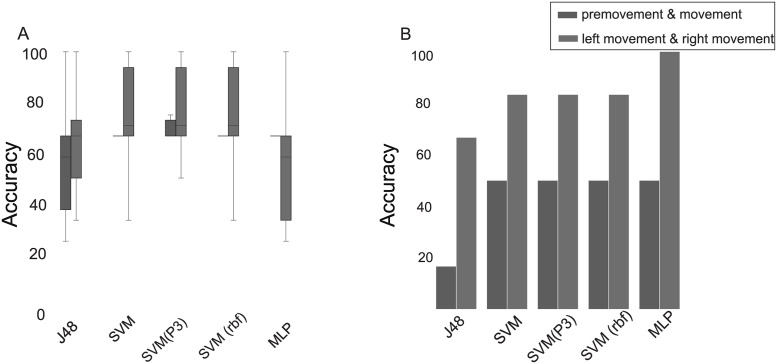
Machine learning-based model performance. (A) Training accuracies for central regions on using SVM had outperformed MLP and DT for left and right hand movement (SVM: Support Vector machine; SVM-(P3): support vector machine polynomial degree 3, SVM (rbf): Support vector machine—radial basis function, MLP: Multilayer Perceptron) (B) Testing accuracy performance with central regions SVMs has performed similarly with 50% accuracy for premovement & movement, and 80% accuracy for left and right hand movement classification DT has performed moderately for left vs right hand movement.

The premovement and movement tasks dataset acquired from central regions (50 training samples) allowed to generate a statistically significant model of classification with 70% training accuracy (see Fig 6A) and 50% (6 samples) test accuracy (see Fig 6B) using SVM and MLP algorithms. In the case of the left- and right-hand movement 76% training accuracy and 83% (F1 = 0.83; AUC = 1) test accuracy with the SVM polynomial kernel. (See S6 and S7 Tables in S1 File for accuracy, AUC and F1 scores) was observed. For the 20-features dataset generated with feature ranking methods, a 16% error rate was obtained when using decision trees and by pruning the ranked 20 features to 5 leaf nodes, and with minimum samples to 3 (see S5 Fig in S1 File for accuracy with 20 ranked features dataset).

## 4. Discussion

In this study, an exploration of grasped arm movement dissecting the pre-movement and movement-specific MRCP and ERD/ERS morphology was performed in the context of multiple movement tasks. Interpreting EEG underlying left or right arm movement, allowed learning models to discriminate movement intention or execution for clinically relevant assessments and can be used for current and future brain-machine interfaces. Specifically, in this study, feature-based interpretations of grasped movement and premovement have been highlighted within EEG data using the underlying low-frequency time-domain data characteristics & from the γ oscillations observed in the central cortical regions.

The combination of the temporal peak of the readiness potential peak and the μ, β, and γ oscillations can be used to precisely represent premovement and grasped movement tasks. The signal’s temporal shift in deflection towards the positive before the onset of movement can be used as a potential biomarker to differentiate movement intention and grasped movement. Central regions’ importance in controlling arm movements may be justified as the accuracy reported among those electrodes was higher.

The main differences between premovement and movement may be observed within the first 0.5 s before the movement onset, mainly over the contralateral primary motor cortex (locations C3, C4). Our results suggest that the attenuated α oscillations and increased β oscillation topographies at central and parietal regions were indicative of hand movement states. A decrease in α, β, and γ-band activity compared to the “relax” (resting-state) task could be indicative of the progression of the grasped movement, and a rebound β and γ frequency activity after grasped movement can serve as spectral biomarkers of pre and post-movement assessments. In the C3 and C4 electrode regions, other than the μ and β bands, the γ band was also associated with grasped movement. Pre-movement and grasped movement conditions were significantly different in the central regions implying that the central regions may provide optimal features to train and predict BCI-related classification models. Although some of the frontal electrodes were evaluated, better accuracy observed with C3-C4 electrodes in the central regions corresponds to the capacity of discrimination of left- and right-hand movement by these overlapping hand regions. The β and γ-band variations across asymmetry may be crucial for discriminating left- and right-hand grasped movement and the pre-movement suppression and post-grasped movement rebound of β band in the context of the laterality of neural activity especially in the ipsilateral motor cortical areas, which could be relevant for the task-based evaluation of healthy and Parkinson’s disease patients.

Through machine learning analysis, model-based discriminators were generated focusing on premovement and grasped movement task classification. Machine learning classifiers could discriminate left and right direction movement using the same features as in premovement and movement tasks. Improved classification accuracy while discriminating pre-movement and grasped movement, was obtained using peak amplitude of readiness potential and the PSD estimates of μ, β, and γ oscillations. In this study, SVM with a polynomial kernel with a lower order provided the highest accuracy over other machine learning classifiers, and allowed faster model building and testing while discriminating peri-movement and movement data. MLP also performed relatively well, although not as optimally as the SVM compared to several other interpretable classifiers on the EEG datasets. In the case of reducing features, combining decision tree-based feature ranking and applying pruning as pre-processing of data could improve classification accuracy. Estimating across several EEG-based tasks and measurement modalities as the pruning could help generate a more generic learning model that may need to be tested on movement imagery and movement execution-based arm movement datasets.

The artifact removal in EEG involved the removal of eye-blink, eye movements, and tongue movements helped augment the classifier’s accuracy and was crucial to remove those data points that had EEG artifacts before evaluating with classifiers. The study also suggests that employing low-cost consumer-grade EEG devices, given their ease of integration and instead of and alongside clinical-grade devices, could capture critical information related to grasped movement and its execution had similar discrimination models and may be valuable in finding candidates for clinical trials.

## 5. Conclusion

Premovement and grasped movement may vary across spatio-temporal scales but discrimination of left and right grasped movement could be performed with temporal and spectral analysis and combining classification methods for decoding pre-movement neural activity in the case of stereotyped left or right arm movements. Interpretation relied on low-frequency time-domain signals and γ oscillations for decoding the left and right movement tasks and with minor variations across genders. As observed in neurological conditions, activations in ipsilateral sensorimotor areas can be used to interpret compensation mechanisms related to the movement implying bi-electrode C3-C4 regions may be task discriminative. Although not presented in this study, the temporal and spectral features can be the foundation for novel control strategies targeting the directional choices of an operational robotic arm.

## Supporting information

S1 File (PDF)

## References

[1] Marquez-Chin C, Popovic MR. Functional electrical stimulation therapy for restoration of motor function after spinal cord injury and stroke: a review. Biomed Eng Online. 2020;19: 34. doi: 10.1186/s12938-020-00773-4 32448143 PMC7245767

[2] López-Larraz E, Montesano L, Gil-Agudo Á, Minguez J. Continuous decoding of movement intention of upper limb self-initiated analytic movements from pre-movement EEG correlates. J Neuroeng Rehabil. 2014;11: 153. doi: 10.1186/1743-0003-11-153 25398273 PMC4247645

[3] Green JB, Sora E, Bialy Y, Ricamato A, Thatcher RW. Cortical motor reorganization after paraplegia: an EEG study. Neurology. 1999;53: 736–43. doi: 10.1212/wnl.53.4.736 10489034

[4] Antelis JM, Montesano L, Ramos-Murguialday A, Birbaumer N, Minguez J. Continuous decoding of intention to move from contralesional hemisphere brain oscillations in severely affected chronic stroke patients. Annu Int Conf IEEE Eng Med Biol Soc IEEE Eng Med Biol Soc Annu Int Conf. 2012;2012: 4099–103. doi: 10.1109/EMBC.2012.6346868 23366829

[5] Morash V, Bai O, Furlani S, Lin P, Hallett M. Classifying EEG signals preceding right hand, left hand, tongue, and right foot movements and motor imageries. Clin Neurophysiol. 2008;119: 2570–2578. doi: 10.1016/j.clinph.2008.08.013 18845473 PMC2602863

[6] Buch E, Weber C, Cohen LG, Braun C, Dimyan MA, Ard T, et al. Think to move: a neuromagnetic brain-computer interface (BCI) system for chronic stroke. Stroke. 2008;39: 910–7. doi: 10.1161/STROKEAHA.107.505313 18258825 PMC5494966

[7] Lew E, Chavarriaga R, Silvoni S, Millán J del R. Detection of self-paced reaching movement intention from EEGsignals. Front Neuroeng. 2012;5. doi: 10.3389/FNENG.2012.00013 23055968 PMC3458432

[8] Wolpaw JR, Editor G, Birbaumer N, Heetderks WJ, Mcfarland DJ, Peckham PH, et al. Brain–Computer Interface Technology: A Review of the First International Meeting. 2000;8: 164–173. 10.1109/tre.2000.847807 10896178

[9] Daly JJ, Wolpaw JR. Brain-computer interfaces in neurological rehabilitation. Lancet Neurol. 2008;7: 1032–43. doi: 10.1016/S1474-4422(08)70223-0 18835541

[10] Lebedev MA, Nicolelis MAL. Brain-machine interfaces: past, present and future. Trends Neurosci. 2006;29: 536–46. doi: 10.1016/j.tins.2006.07.004 16859758

[11] López-Larraz E, Antelis JM, Montesano L, Gil-Agudo A, Minguez J. Continuous decoding of motor attempt and motor imagery from EEG activity in spinal cord injury patients. Annu Int Conf IEEE Eng Med Biol Soc IEEE Eng Med Biol Soc Annu Int Conf. 2012;2012: 1798–801. doi: 10.1109/EMBC.2012.6346299 23366260

[12] Iyengar V, Santos MJ, Ko M, Aruin AS. Grip force control in individuals with multiple sclerosis. Neurorehabil Neural Repair. 2009;23: 855–61. doi: 10.1177/1545968309338194 19531607

[13] Leocani L, Colombo B, Magnani G, Martinelli-Boneschi F, Cursi M, Rossi P, et al. Fatigue in multiple sclerosis is associated with abnormal cortical activation to voluntary movement—EEG evidence. Neuroimage. 2001;13: 1186–92. doi: 10.1006/nimg.2001.0759 11352624

[14] Paek AY, Agashe HA, Contreras-Vidal JL. Decoding repetitive finger movements with brain activity acquired via non-invasive electroencephalography. Front Neuroeng. 2014;7: 3. doi: 10.3389/fneng.2014.00003 24659964 PMC3952032

[15] Iturrate I, Chavarriaga R, Pereira M, Zhang H, Corbet T, Leeb R, et al. Human EEG reveals distinct neural correlates of power and precision grasping types. Neuroimage. 2018;181: 635–644. doi: 10.1016/j.neuroimage.2018.07.055 30056196

[16] Schwarz A, Ofner P, Pereira J, Sburlea AI, Müller-Putz GR. Decoding natural reach-and-grasp actions from human EEG. J Neural Eng. 2018;15: 016005. doi: 10.1088/1741-2552/aa8911 28853420

[17] Kim YJ, Park SW, Yeom HG, Bang MS, Kim JS, Chung CK, et al. A study on a robot arm driven by three-dimensional trajectories predicted from non-invasive neural signals. Biomed Eng Online. 2015;14: 81. doi: 10.1186/s12938-015-0075-8 26290069 PMC4545996

[18] Ofner P, Schwarz A, Pereira J, Wyss D, Wildburger R, Müller-Putz GR. Attempted Arm and Hand Movements can be Decoded from Low-Frequency EEG from Persons with Spinal Cord Injury. Sci Rep. 2019;9: 7134. doi: 10.1038/s41598-019-43594-9 31073142 PMC6509331

[19] Wagner J, Makeig S, Gola M, Neuper C, Müller-Putz G. Distinct β Band Oscillatory Networks Subserving Motor and Cognitive Control during Gait Adaptation. J Neurosci. 2016;36: 2212–26. doi: 10.1523/JNEUROSCI.3543-15.2016 26888931 PMC6602036

[20] Feige B, Kristeva-Feige R, Rossi S, Pizzella V, Rossini P-M. Neuromagnetic study of movement-related changes in rhythmic brain activity. Brain Res. 1996;734: 252–260. doi: 10.1016/0006-8993(96)00648-8 8896832

[21] Cassim F, Monaca C, Szurhaj W, Bourriez JL, Defebvre L, Derambure P, et al. Does post-movement beta synchronization reflect an idling motor cortex? Neuroreport. 2001;12: 3859–63. doi: 10.1097/00001756-200112040-00051 11726809

[22] Pfurtscheller G, Lopes FH. Event-related EEG / MEG synchronization and desynchronization: basic principles. Clin Neurophysiol. 1999;110: 1842–1857. doi: 10.1016/s1388-2457(99)00141-8 10576479

[23] Pfurtscheller G, Neuper C. Future prospects of ERD/ERS in the context of brain-computer interface (BCI) developments. Prog Brain Res. 2006;159: 433–7. doi: 10.1016/S0079-6123(06)59028-4 17071247

[24] Bai O, Rathi V, Lin P, Huang D, Battapady H, Fei D-Y, et al. Prediction of human voluntary movement before it occurs. Clin Neurophysiol. 2011;122: 364–72. doi: 10.1016/j.clinph.2010.07.010 20675187 PMC5558611

[25] Ibáñez J, Serrano JI, del Castillo MD, Monge-Pereira E, Molina-Rueda F, Alguacil-Diego I, et al. Detection of the onset of upper-limb movements based on the combined analysis of changes in the sensorimotor rhythms and slow cortical potentials. J Neural Eng. 2014;11: 056009. doi: 10.1088/1741-2560/11/5/056009 25082789

[26] Tang Z, Sun S, Zhang S, Chen Y, Li C, Chen S. A Brain-Machine Interface Based on ERD/ERS for an Upper-Limb Exoskeleton Control. Sensors (Basel). 2016;16. doi: 10.3390/s16122050 27918413 PMC5191031

[27] Deecke L, Scheid P, Kornhuber HH. Distribution of readiness potential, pre-motion positivity, and motor potential of the human cerebral cortex preceding voluntary finger movements. Exp brain Res. 1969;7: 158–68. doi: 10.1007/BF00235441 5799432

[28] Kornhuber HH, Deecke L. Brain potential changes in voluntary and passive movements in humans: readiness potential and reafferent potentials. Pflügers Arch—Eur J Physiol. 2016;468: 1115–1124. doi: 10.1007/s00424-016-1852-3 27392465

[29] Shakeel A, Navid MS, Anwar MN, Mazhar S, Jochumsen M, Niazi IK. A Review of Techniques for Detection of Movement Intention Using Movement-Related Cortical Potentials. Comput Math Methods Med. 2015;2015: 1–13. doi: 10.1155/2015/346217 26881008 PMC4735988

[30] Dremstrup K, Gu Y, do Nascimento OF, Farina D. Movement-Related Cortical Potentials and Their Application in Brain-Computer Interfacing. Introduction to Neural Engineering for Motor Rehabilitation. Hoboken, NJ, USA: John Wiley & Sons, Inc.; 2013. pp. 253–266.

[31] Shibasaki H, Hallett M. What is the Bereitschaftspotential? Clin Neurophysiol. 2006;117: 2341–56. doi: 10.1016/j.clinph.2006.04.025 16876476

[32] Libet B, Gleason CA, Wright EW, Pearl DK. Time of Conscious Intention to Act in Relation to Onset of Cerebral Activity (Readiness-Potential). Neurophysiology of Consciousness. Boston, MA: Birkhäuser Boston; 1993. pp. 249–268.

[33] Libet B, Wright E., Gleason C. Readiness-potentials preceding unrestricted ‘spontaneous’ vs. pre-planned voluntary acts. Electroencephalogr Clin Neurophysiol. 1982;54: 322–335. doi: 10.1016/0013-4694(82)90181-x 6179759

[34] Elbert T, Rockstroh B. Threshold regulation—a key to the understanding of the combined dynamics of EEG and event-related potentials. J Psychophysiol. 1987;1: 317–333. Available: https://kops.uni-konstanz.de/handle/123456789/16254

[35] Birbaumer N, Elbert T, Canavan AG, Rockstroh B. Slow potentials of the cerebral cortex and behavior. Physiol Rev. 1990;70: 1–41. doi: 10.1152/physrev.1990.70.1.1 2404287

[36] Yilmaz O, Cho W, Braun C, Birbaumer N, Ramos-Murguialday A. Movement related cortical potentials in severe chronic stroke. Annu Int Conf IEEE Eng Med Biol Soc IEEE Eng Med Biol Soc Annu Int Conf. 2013;2013: 2216–9. doi: 10.1109/EMBC.2013.6609976 24110163

[37] Gu Y, Farina D, Murguialday AR, Dremstrup K, Montoya P, Birbaumer N. Off line identification of imagined speed of wrist movements in paralyzed ALS patients from single-trial EEG. Front Neurosci. 2009;3: 3. 20582286 10.3389/neuro.20.003.2009 PMC2858603

[38] Karimi F, Kofman J, Mrachacz-Kersting N, Farina D, Jiang N. Detection of Movement Related Cortical Potentials from EEG Using Constrained ICA for Brain-Computer Interface Applications. Front Neurosci. 2017;11: 356. doi: 10.3389/fnins.2017.00356 28713232 PMC5492875

[39] Karimi F, Niu J, Almeida Q, Jiang N. Movement Related Cortical Potentials in Parkinson’s Disease Patients with Freezing of Gait. Annu Int Conf IEEE Eng Med Biol Soc IEEE Eng Med Biol Soc Annu Int Conf. 2020;2020: 2857–2860. doi: 10.1109/EMBC44109.2020.9176000 33018602

[40] Sakata H, Taira M. Parietal control of hand action. Curr Opin Neurobiol. 1994;4: 847–56. Available: http://www.ncbi.nlm.nih.gov/pubmed/7888768 7888768 10.1016/0959-4388(94)90133-3

[41] Raos V, Umiltá M-A, Gallese V, Fogassi L. Functional Properties of Grasping-Related Neurons in the Dorsal Premotor Area F2 of the Macaque Monkey. J Neurophysiol. 2004;92: 1990–2002. doi: 10.1152/jn.00154.2004 15163668

[42] Godschalk M. Activity of single neurons in monkey cortex preceding sensory cued limb movements. Electroencephalogr Clin Neurophysiol Suppl. 1991;42: 71–9. Available: http://www.ncbi.nlm.nih.gov/pubmed/1915033 1915033

[43] Moll L, Kuypers HG. Premotor cortical ablations in monkeys: contralateral changes in visually guided reaching behavior. Science. 1977;198: 317–9. Available: http://www.ncbi.nlm.nih.gov/pubmed/410103 410103 10.1126/science.410103

[44] Begliomini C, Caria A, Grodd W, Castiello U. Comparing Natural and Constrained Movements: New Insights into the Visuomotor Control of Grasping. Robertson E, editor. PLoS One. 2007;2: e1108. doi: 10.1371/journal.pone.0001108 17971871 PMC2040199

[45] Begliomini C, Nelini C, Caria A, Grodd W, Castiello U. Cortical Activations in Humans Grasp-Related Areas Depend on Hand Used and Handedness. Warrant E, editor. PLoS One. 2008;3: e3388. doi: 10.1371/journal.pone.0003388 18846222 PMC2561002

[46] Culham JC, Danckert SL, De Souza JFX, Gati JS, Menon RS, Goodale MA. Visually guided grasping produces fMRI activation in dorsal but not ventral stream brain areas. Exp Brain Res. 2003;153: 180–189. doi: 10.1007/s00221-003-1591-5 12961051

[47] Dohle C, Ostermann G, Hefter H, Freund HJ. Different coupling for the reach and grasp components in bimanual prehension movements. Neuroreport. 2000;11: 3787–91. Available: http://www.ncbi.nlm.nih.gov/pubmed/11117492 11117492 10.1097/00001756-200011270-00039

[48] Frey SH, Gerry VE. Modulation of Neural Activity during Observational Learning of Actions and Their Sequential Orders. J Neurosci. 2006;26: 13194–13201. doi: 10.1523/JNEUROSCI.3914-06.2006 17182769 PMC6674989

[49] Culham JC, Valyear KF. Human parietal cortex in action. Curr Opin Neurobiol. 2006;16: 205–212. doi: 10.1016/j.conb.2006.03.005 16563735

[50] Nelissen K, Vanduffel W. Grasping-related functional magnetic resonance imaging brain responses in the macaque monkey. J Neurosci. 2011;31: 8220–9. doi: 10.1523/JNEUROSCI.0623-11.2011 21632943 PMC3117146

[51] Friedman RM, Chehade NG, Roe AW, Gharbawie OA. Optical imaging reveals functional domains in primate sensorimotor cortex. Neuroimage. 2020;221: 117188. doi: 10.1016/j.neuroimage.2020.117188 32711067 PMC7841645

[52] Functional brain imaging based on ERD/ERS. Vision Res. 2001;41: 1257–1260. doi: 10.1016/s0042-6989(00)00235-2 11322970

[53] Diwakar S, Bodda S, Nutakki C, Vijayan A, Achuthan K. Neural Control using EEG as a BCI Technique for Low Cost Prosthetic Arms. Proceedings of the International Conference on Neural Computation Theory and Applications (NCTA-2014). Rome, Italy: SCITEPRESS (Science and Technology Publications, Lda); 2014. pp. 270–275.

[54] Bodda S, Chandranpillai H, Viswam P, Krishna S, Nair B, Diwakar S. Categorizing Imagined Right and Left Motor Imagery BCI Tasks for Low-cost Robotic Neuroprosthesis. Proceedings of ICEEOT 2016. 2016. pp. 1–4.

[55] Pfurtscheller G, Brunner C, Schlögl A, Lopes da Silva FH. Mu rhythm (de)synchronization and EEG single-trial classification of different motor imagery tasks. Neuroimage. 2006;31: 153–9. doi: 10.1016/j.neuroimage.2005.12.003 16443377

[56] Pfurtscheller G, Neuper C, Flotzinger D, Pregenzer M. EEG-based discrimination between imagination of right and left hand movement. Electroencephalogr Clin Neurophysiol. 1997;103: 642–51. doi: 10.1016/s0013-4694(97)00080-1 9546492

[57] McFarland DJ, Miner L a, Vaughan TM, Wolpaw JR. Mu and beta rhythm topographies during motor imagery and actual movements. Brain Topogr. 2000;12: 177–86. doi: 10.1023/a:1023437823106 10791681

[58] Tariq M, Trivailo PM, Simic M. Mu-Beta event-related (de)synchronization and EEG classification of left-right foot dorsiflexion kinaesthetic motor imagery for BCI. PLoS One. 2020;15: e0230184. doi: 10.1371/journal.pone.0230184 32182270 PMC7077852

[59] Tsui CSL, Gan JQ, Hu H. A self-paced motor imagery based brain-computer interface for robotic wheelchair control. Clin EEG Neurosci. 2011;42: 225–9. doi: 10.1177/155005941104200407 22208119

[60] Huang Dandan, Qian Kai, Fei Ding-Yu, Jia Wenchuan, Chen Xuedong, Bai Ou. Electroencephalography (EEG)-Based Brain–Computer Interface (BCI): A 2-D Virtual Wheelchair Control Based on Event-Related Desynchronization/Synchronization and State Control. IEEE Trans Neural Syst Rehabil Eng. 2012;20: 379–388. doi: 10.1109/TNSRE.2012.2190299 22498703

[61] Magnani G, Cursi M, Volontc TA. Event-Related Desynchronization to Contingent Negative Variation and Self-Paced Movement Paradigms in Parkinson ‘ s Disease. Mov Disord. 1998;13: 653–660. doi: 10.1002/mds.870130408 9686770

[62] Ohara S, Ikeda A, Kunieda T, Yazawa S, Baba K, Nagamine T, et al. Movement-related change of electrocorticographic activity in human supplementary motor area proper. Brain. 2000;123: 1203–1215. doi: 10.1093/brain/123.6.1203 10825358

[63] Sheer DE, Grandstaff NW, Benignus VA. Behavior and 40-c-sec. electrical activity in the brain. Psychol Rep. 1966;19: 1333–4. doi: 10.2466/pr0.1966.19.3f.1333 5956433

[64] C NE, M DL, G B, L RP. Functional Mapping of Human Sensorimotor Cortex With Electrocorticographic Spectral Analysis. II. Event-related Synchronization in the Gamma Band. Brain. 1998;121 (Pt 1. doi: 10.1093/BRAIN/121.12.2301 9874481

[65] Quandt F, Reichert C, Hinrichs H, Heinze HJ, Knight RT, Rieger JW. Single trial discrimination of individual finger movements on one hand: a combined MEG and EEG study. Neuroimage. 2012;59: 3316–24. doi: 10.1016/j.neuroimage.2011.11.053 22155040 PMC4028834

[66] Agashe HA, Contreras-Vidal JL. Reconstructing hand kinematics during reach to grasp movements from electroencephalographic signals. 2011 Annual International Conference of the IEEE Engineering in Medicine and Biology Society. IEEE; 2011. pp. 5444–5447. 10.1109/IEMBS.2011.6091389 22255569

[67] Szurhaj W, Labyt E, Bourriez J-L, Kahane P, Chauvel P, Mauguière F, et al. Relationship between intracerebral gamma oscillations and slow potentials in the human sensorimotor cortex. Eur J Neurosci. 2006;24: 947–54. doi: 10.1111/j.1460-9568.2006.04876.x 16930422

[68] Pfurtscheller G, Graimann B, Huggins JE, Levine SP, Schuh LA. Spatiotemporal patterns of beta desynchronization and gamma synchronization in corticographic data during self-paced movement. Clin Neurophysiol. 2003;114: 1226–36. doi: 10.1016/s1388-2457(03)00067-1 12842719

[69] Brovelli A, Lachaux J-P, Kahane P, Boussaoud D. High gamma frequency oscillatory activity dissociates attention from intention in the human premotor cortex. Neuroimage. 2005;28: 154–64. doi: 10.1016/j.neuroimage.2005.05.045 16023374

[70] Ramos-Murguialday A, Birbaumer N. Brain oscillatory signatures of motor tasks. J Neurophysiol. 2015;113: 3663–82. doi: 10.1152/jn.00467.2013 25810484 PMC4468978

[71] Ball T, Demandt E, Mutschler I, Neitzel E, Mehring C, Vogt K, et al. Movement related activity in the high gamma range of the human EEG. Neuroimage. 2008;41: 302–10. doi: 10.1016/j.neuroimage.2008.02.032 18424182

[72] Titgemeyer Y, Surges R, Altenmüller D-M, Fauser S, Kunze A, Lanz M, et al. Can commercially available wearable EEG devices be used for diagnostic purposes? An explorative pilot study. Epilepsy Behav. 2020;103: 106507. doi: 10.1016/j.yebeh.2019.106507 31645318

[73] Kamousi B, Grant AM, Bachelder B, Yi J, Hajinoroozi M, Woo R. Comparing the quality of signals recorded with a rapid response EEG and conventional clinical EEG systems. Clin Neurophysiol Pract. 2019;4: 69–75. doi: 10.1016/j.cnp.2019.02.002 30976727 PMC6444024

[74] Ratti E, Waninger S, Berka C, Ruffini G, Verma A. Comparison of Medical and Consumer Wireless EEG Systems for Use in Clinical Trials. Front Hum Neurosci. 2017;11: 398. doi: 10.3389/fnhum.2017.00398 28824402 PMC5540902

[75] Pistohl T, Schulze-Bonhage A, Aertsen A, Mehring C, Ball T. Decoding natural grasp types from human ECoG. Neuroimage. 2012;59: 248–60. doi: 10.1016/j.neuroimage.2011.06.084 21763434

[76] Pistohl T, Schmidt TSB, Ball T, Schulze-Bonhage A, Aertsen A, Mehring C. Grasp detection from human ECoG during natural reach-to-grasp movements. PLoS One. 2013;8: e54658. doi: 10.1371/journal.pone.0054658 23359537 PMC3554656

[77] Agashe HA, Paek AY, Zhang Y, Contreras-Vidal JL. Global cortical activity predicts shape of hand during grasping. Front Neurosci. 2015;9: 121. doi: 10.3389/fnins.2015.00121 25914616 PMC4391035

[78] Agashe HA, Paek AY, Contreras-Vidal JL. Multisession, noninvasive closed-loop neuroprosthetic control of grasping by upper limb amputees. Prog Brain Res. 2016;228: 107–28. doi: 10.1016/bs.pbr.2016.04.016 27590967

[79] Randazzo L, Iturrate I, Chavarriaga R, Leeb R, Del Millan JR. Detecting intention to grasp during reaching movements from EEG. Annu Int Conf IEEE Eng Med Biol Soc IEEE Eng Med Biol Soc Annu Int Conf. 2015;2015: 1115–8. doi: 10.1109/EMBC.2015.7318561 26736461

[80] Jochumsen M, Niazi IK, Dremstrup K, Kamavuako EN. Detecting and classifying three different hand movement types through electroencephalography recordings for neurorehabilitation. Med Biol Eng Comput. 2016;54: 1491–501. doi: 10.1007/s11517-015-1421-5 26639017

[81] Grünewald G, Grünewald-Zuberbier E, Netz J, Hömberg V, Sander G. Relationships between the late component of the contingent negative variation and the bereitschaftspotential. Electroencephalogr Clin Neurophysiol. 1979;46: 538–545. doi: 10.1016/0013-4694(79)90007-5 88342

[82] Toro C, Deuschl G, Thatcher R, Sato S, Kufta C, Hallett M. Event-related desynchronization and movement-related cortical potentials on the ECoG and EEG. Electroencephalogr Clin Neurophysiol. 1994;93: 380–9. doi: 10.1016/0168-5597(94)90126-0 7525246

[83] Nagamine T, Kajola M, Salmelin R, Shibasaki H, Hari R. Movement-related slow cortical magnetic fields and changes of spontaneous MEG- and EEG-brain rhythms. Electroencephalogr Clin Neurophysiol. 1996;99: 274–286. doi: 10.1016/0013-4694(96)95154-8 8862117

[84] Babiloni C, Carducci F, Cincotti F, Rossini PM, Neuper C, Pfurtscheller G, et al. Human movement-related potentials vs desynchronization of EEG alpha rhythm: a high-resolution EEG study. Neuroimage. 1999;10: 658–65. doi: 10.1006/nimg.1999.0504 10600411

[85] Babiloni C, Babiloni F, Carducci F, Cincotti F, Del Percio C, Hallett M, et al. Chapter 55 High resolution EEG of sensorimotor brain functions: mapping ERPs or mu ERD? Suppl Clin Neurophysiol. 2002;54: 365–371. doi: 10.1016/S1567-424X(09)70475-1

[86] Jeannerod M. The representing brain: Neural correlates of motor intention and imagery. Behav Brain Sci. 1994;17: 187. doi: 10.1017/S0140525X00034026

[87] Gramfort A, Luessi M, Larson E, Engemann DA, Strohmeier D, Brodbeck C, et al. MEG and EEG data analysis with MNE-Python. Front Neurosci. 2013;7: 1–13. 24431986 10.3389/fnins.2013.00267 PMC3872725

[88] de Cheveigné A, Nelken I. Filters: When, Why, and How (Not) to Use Them. Neuron. 2019;102: 280–293. doi: 10.1016/j.neuron.2019.02.039 30998899

[89] de Cheveigné A, Arzounian D. Robust detrending, rereferencing, outlier detection, and inpainting for multichannel data. Neuroimage. 2018;172: 903–912. doi: 10.1016/j.neuroimage.2018.01.035 29448077 PMC5915520

[90] Widmann A, Schröger E, Maess B. Digital filter design for electrophysiological data–a practical approach. J Neurosci Methods. 2015;250: 34–46. doi: 10.1016/j.jneumeth.2014.08.002 25128257

[91] Widmann A, Schröger E. Filter effects and filter artifacts in the analysis of electrophysiological data. Front Psychol. 2012;3: 233. doi: 10.3389/fpsyg.2012.00233 22787453 PMC3391960

[92] Hyvärinen A, Oja E. Independent component analysis: algorithms and applications. Neural Networks. 2000;13: 411–430. doi: 10.1016/s0893-6080(00)00026-5 10946390

[93] Bell AJ, Sejnowski TJ. An information-maximization approach to blind separation and blind deconvolution. Neural Comput. 1995;7: 1129–59. doi: 10.1162/neco.1995.7.6.1129 7584893

[94] Linsker R. An Application of the Principle of Maximum Information Preservation to Linear Systems. Adv Neural Inf Process Syst. 1988;1. https://proceedings.neurips.cc/paper/1988/hash/ec8956637a99787bd197eacd77acce5e-Abstract.html

[95] Slepian D. Prolate Spheroidal Wave Functions, Fourier Analysis, and Uncertainty-V: The Discrete Case. Bell Syst Tech J. 1978;57: 1371–1430. doi: 10.1002/j.1538-7305.1978.tb02104.x

[96] Thomson DJ. Spectrum estimation and harmonic analysis. Proc IEEE. 1982;70: 1055–1096. doi: 10.1109/PROC.1982.12433

[97] Thomson D. Jackknifing Multitaper Spectrum Estimates. IEEE Signal Process Mag. 2007;24: 20–30. doi: 10.1109/MSP.2007.4286561

[98] Wright DJ, Holmes PS, Smith D. Using the movement-related cortical potential to study motor skill learning. J Mot Behav. 2011;43: 193–201. doi: 10.1080/00222895.2011.557751 21462065

[99] Motulsky GraphPAD Software Inc. (Firm), H. GraphPAD Prism 7.04: scientific graphing, curve fitting & statistics. San Diego, Calif.: GraphPAD Software Inc.; 2002.

[100] Utgoff PE. Incremental Induction of Decision Trees. Mach Learn. 1989;4: 161–186. doi: 10.1023/A:1022699900025

[101] Quinlan JR. Learning Efficient Classification Procedures and Their Application to Chess End Games. Machine Learning. Berlin, Heidelberg: Springer Berlin Heidelberg; 1983. pp. 463–482.

[102] Wagner HM. Principles of operations research, with applications to managerial decisions. Prentice-Hall; 1969.

[103] Platt JC, Platt JC. Probabilistic Outputs for Support Vector Machines and Comparisons to Regularized Likelihood Methods. Adv LARGE MARGIN Classif. 1999; 61–74. http://citeseer.ist.psu.edu/viewdoc/summary?doi=10.1.1.41.1639

[104] Vapnik V, Vapnik V, Golowich SE, Smola A. Support Vector Method for Function Approximation, Regression Estimation, and Signal Processing. Adv NEURAL Inf Process Syst 9. 1996;9: 281–287. Available: http://citeseerx.ist.psu.edu/viewdoc/summary?doi=10.1.1.41.3139

[105] Rosenblatt F. Principles of neurodynamics. perceptrons and the theory of brain mechanisms. 1961.

[106] Van Der Malsburg C. Frank Rosenblatt: Principles of Neurodynamics: Perceptrons and the Theory of Brain Mechanisms. Brain Theory. Berlin, Heidelberg: Springer Berlin Heidelberg; 1986. pp. 245–248.

[107] Chang Y-W, Hsieh C-J, Chang K-W, Ringgaard M, Lin C-J. Training and Testing Low-degree Polynomial Data Mappings via Linear SVM. J Mach Learn Res. 2010;11: 1471–1490. Available: http://jmlr.org/papers/v11/chang10a.html

[108] Vert JP, Tsuda K, Schölkopf B. A Primer on Kernel Methods. Kernel Methods Comput Biol 35–70. 2004.

[109] Vapnik V. Pattern recognition using generalized portrait method. Autom Remote Control. 1963;24: 774–780.

[110] Pedregosa F, Varoquaux G, Gramfort A, Michel V, Thirion B, Grisel O, et al. Scikit-learn: Machine Learning in Python. 2012 [cited 17 Aug 2021]. http://arxiv.org/abs/1201.0490

[111] Wilcoxon F. Individual Comparisons by Ranking Methods. Biometrics Bull. 1945;1: 80. doi: 10.2307/3001968

[112] Sarkar C, Cooley S, Srivastava J. Robust Feature Selection Technique using Rank Aggregation. Appl Artif Intell. 2014;28: 243–257. doi: 10.1080/08839514.2014.883903 24839351 PMC4019401

[113] Álvarez JD, Matias-Guiu JA, Cabrera-Martín MN, Risco-Martín JL, Ayala JL. An application of machine learning with feature selection to improve diagnosis and classification of neurodegenerative disorders. BMC Bioinformatics. 2019;20: 491. doi: 10.1186/s12859-019-3027-7 31601182 PMC6788103

