# Supplementary material for: Exploring EEG spectral and temporal dynamics underlying a hand grasp movement
Source: PLoS One. 2022 Jun 23;17(6):e0270366. doi: 10.1371/journal.pone.0270366 (PMC9223346; doi:10.1371/journal.pone.0270366)
Supplement: S1 File — (PDF) [file pone.0270366.s001.pdf]

Exploring EEG spectral and temporal dynamics underlying a hand grasp movement

Sandeep Bodda[1], Shyam Diwakar[*,1,2]

[1]Amrita Mind Brain Center, Amrita Vishwa Vidyapeetham, Amritapuri Campus, Clappana P.O, Kollam, Kerala, India-690525

[2]Department of Electronics and Communication Engineering, School of Engineering, Amrita Vishwa Vidyapeetham, Amritapuri Campus, Clappana P.O, Kollam, Kerala, India-690525

*Corresponding author

Email: shyam@amrita.edu

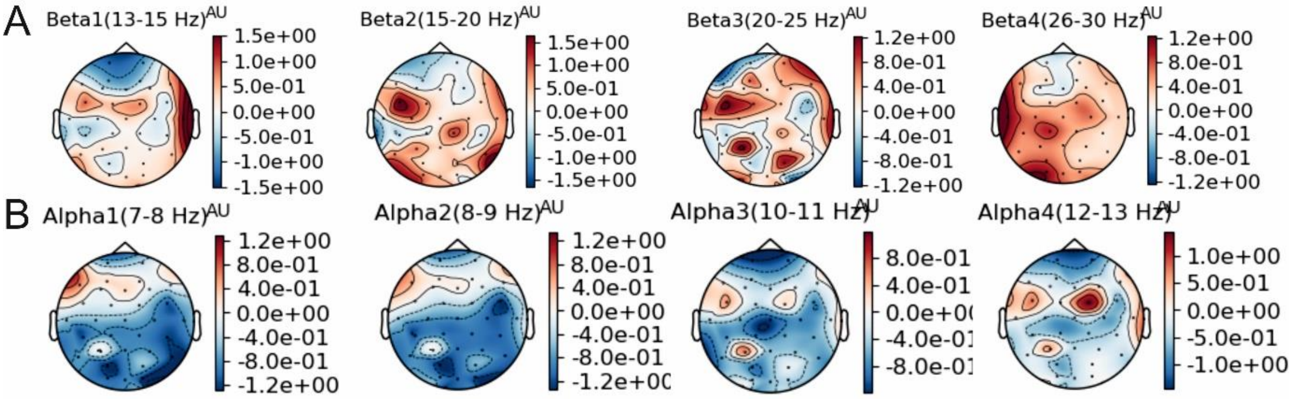

S1 Fig. Increased beta rhythms during movement- ERD/ERS topography maps A: Spectral plot of β sub band regions from 13-30 Hz, sub band regions of 15-25 Hz have shown central regions high activity during movement. B: α/μ modulations spectral plot from 7-11 Hz has shown attenuated activity over different sub band regions of alpha activity across central and parietal regions. (Blue – attenuated activity, red- enhanced activity in the corresponding regions).

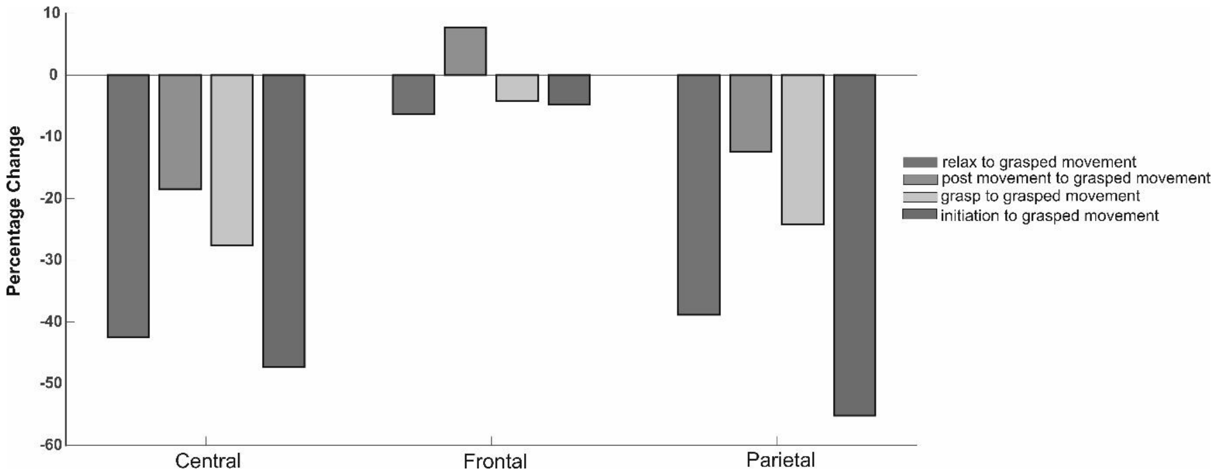

S2 Fig. Decreased theta oscillations in the central regions during grasp to grasped movement Theta oscillations show 20% decrease in central regions and 30% decrease in parietal regions from grasp to grasped movement and 7% increase after the movement in frontal region. (X-axis: Electrode regions, Y-axis: Percentage change in theta oscillations)

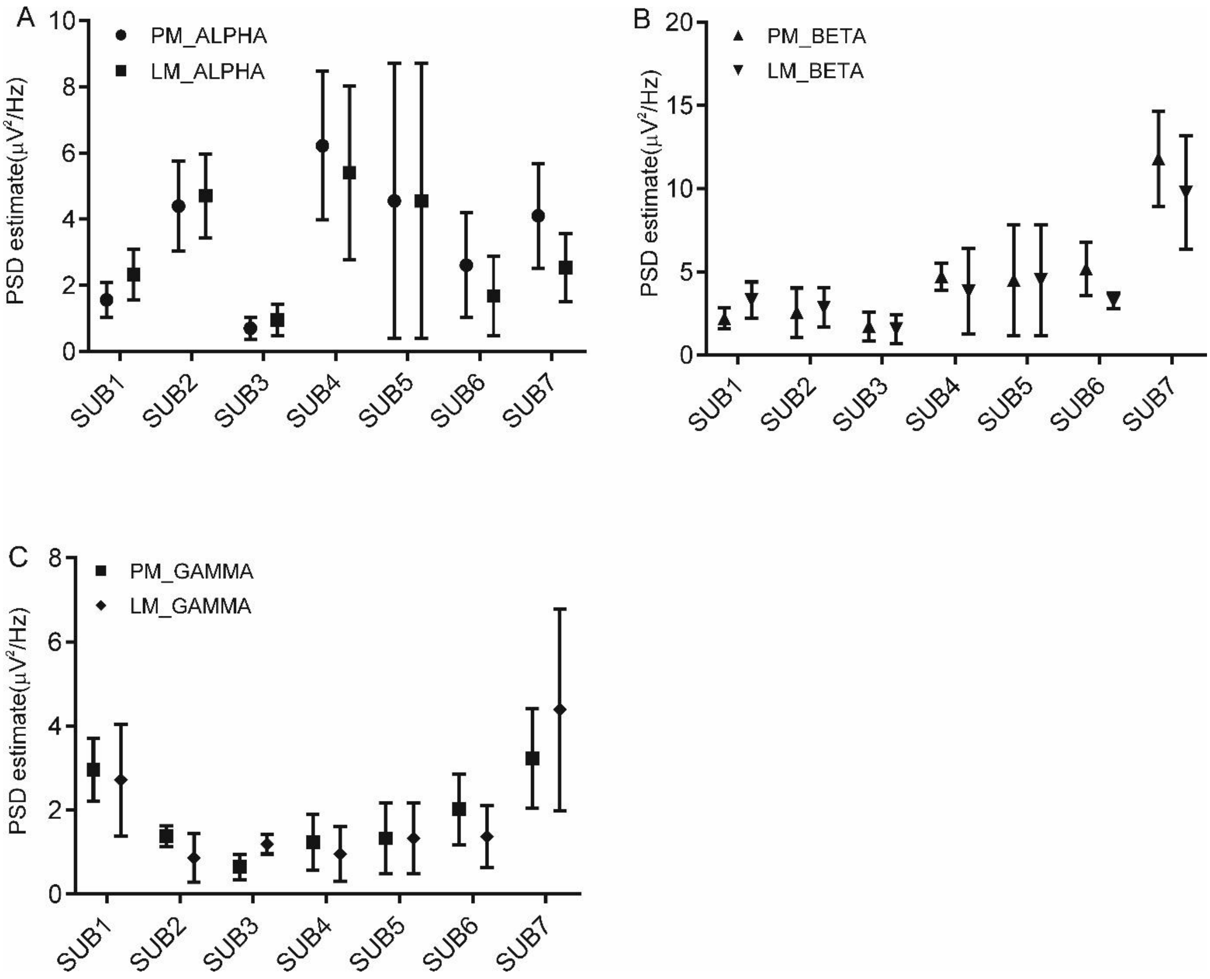

S3 Fig. Inter subject variability for premovement and grasped left movement task (A) α oscillations of premovement task (p value 0.0108), and left movement task (p value 0.0068) across the subjects were statistically significant and different from each other (B) β oscillations for the tasks premovement and left movement are also independent across the subjects with (p value 0.0088 for premovement and 0.0403 for left movement) and (C) γ oscillations show statistical significance with (p value 0.0165 for premovement and 0.0436 for left movement) (X-axis : Subjects Y-axis: PSD estimate)

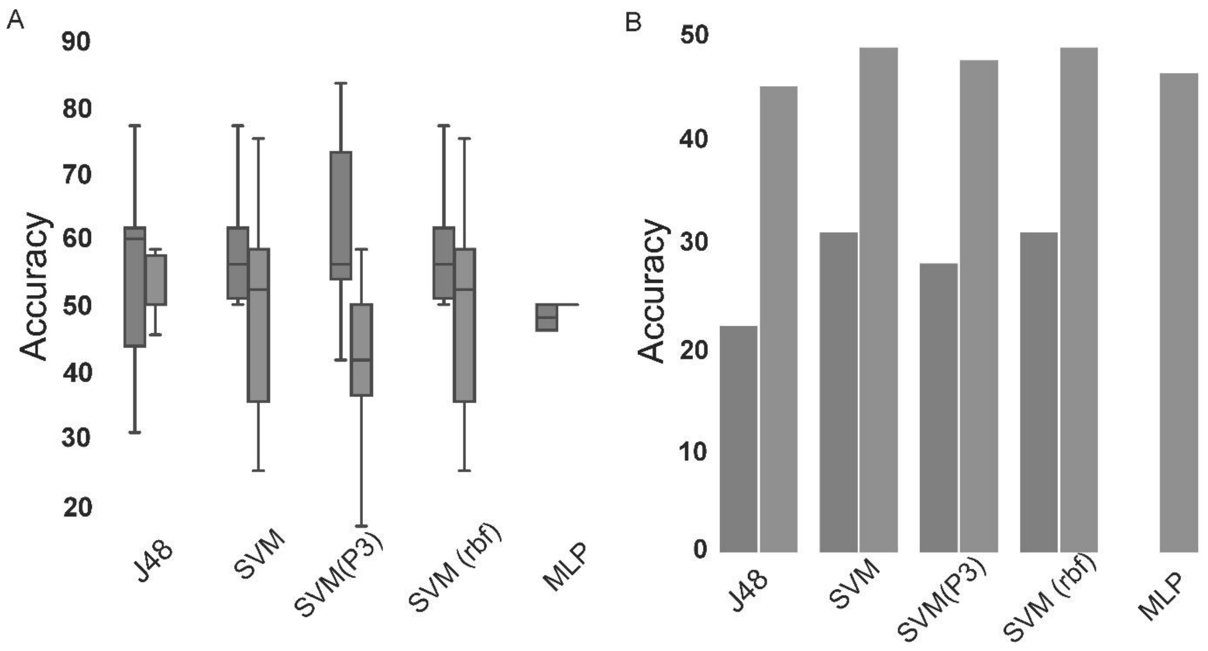

S4 Fig. Machine learning-based model performance over Frontal regions. A: Training accuracy performance for the tasks premovement Vs movement and left movement Vs right movement, SVM (p3) has outperformed the accuracy compared to other algorithms like SVM (rbf) with 57% accuracy and MLP with 48% accuracy for premovement and movement (B) Test dataset accuracy performance for the 14-electrode dataset SVM(p3) performed highest accuracy of 54% whereas MLP with 52% test accuracy and DT has performed route learning (X-axis: machine learning algorithms, Y-axis: accuracy percentage)

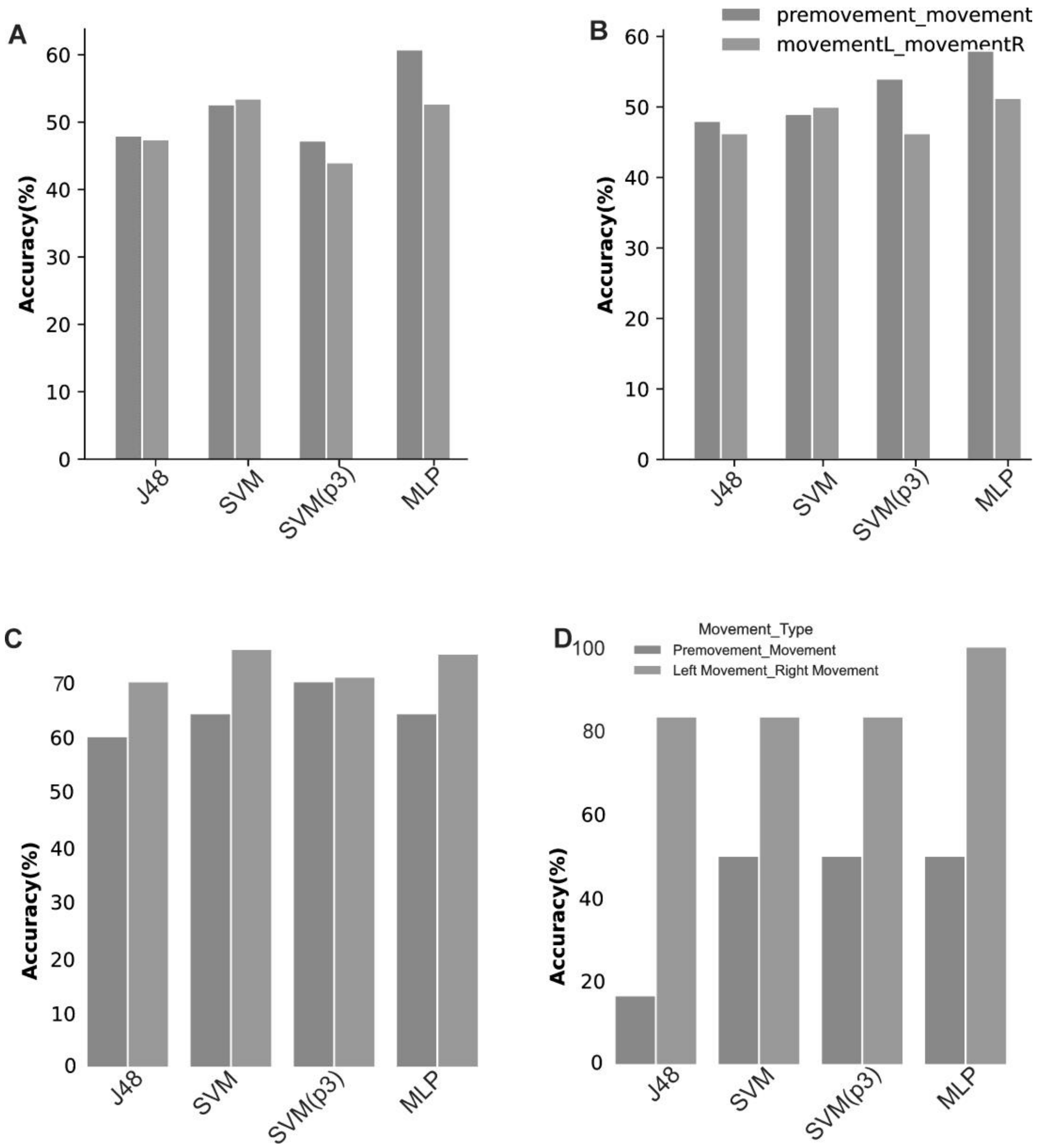

S5 Fig. Model performance for ranked features (A)Training accuracy performance for the tasks premovement Vs movement and left movement Vs right movement, MLP has outperformed with 60% accuracy compared to other for premovement and movement (B) Test dataset accuracy performance for the 14-electrode dataset MLP performed highest accuracy of 58% for premovement and movement whereas for left movement and right movement with 50% accuracy (C) Training accuracy with central regions MLP has outperformed for left and right hand movement with 70% accuracy and 60% accuracy for premovement and movement (D) Testing accuracy performance with central regions MLP, SVM linear and nonlinear has performed similarly for premovement and movement whereas left vs right hand movement SVM performed 80%accuracy (SVM: Support Vector machine; SVM-(P3): support vector machine polynomial degree 3, SVM (rbf): Support vector machine - radial basis function, MLP: Multilayer Perceptron) (X-axis: machine learning algorithms, Y-axis: accuracy percentage)

S1 Table. Average electrode locations on the scalp mapped anatomically labelled region [1]and associate function.

Electrode regions	Underlying Brain Regions	Associate function
C3	Postcentral gyrus, Precentral gyrus	Movement [2,3]
C4	Postcentral gyrus, Precentral gyrus	Movement
F3	Medial Frontal Gyrus (MFG), frontal pole	High level executive functions, decision related process[4],
F4	Frontal pole, MFG	High level executive functions, decision related process,
P3	Superior Lateral Occipital cortex	Shape perception, object recognition [5,6]
P4	Superior Lateral Occipital cortex	Shape perception, object recognition
O1	Occipital pole	Visual perception and processing[7–9]
O2	Occipital pole	Visual perception and processing

S2 Table. Significance test table for premovement, left grasped movement, right grasped movement tasks across α, β, and γ oscillations.

Significance test for the above tabulated data	
**P value and statistical significance**	
Test	Chi-square
Chi-square, df	21.51, 4
P value	0.0003
P value summary	***
One- or two-sided	NA
Statistically significant (P < 0.05)?	Yes
**Data analyzed**	
Number of rows	3
Number of columns	3

S3 Table. Significance test --ANOVA table for male and female genders for the tasks premovement, left grasped movement, right grasped movement tasks and respective frequency-based oscillation bands (α, β, and γ rhythms)

**Two-way ANOVA – Ordinary (Alpha =0.05)**					
**Source of Variation**	**% of total variation**	**P value**	**P value summary**	**Significant?**	
Interaction	5.597	0.0276	*	Yes	
Frequency Oscillations	7.076	<0.0001	****	Yes	
Gender-based tasks	34.05	<0.0001	****	Yes	
**ANOVA table**	**SS**	**DF**	**MS**	**F (DFn, DFd)**	**P value**
Interaction	294.8	10	29.48	F (10, 198) = 2.08	P=0.0276
Gender based tasks	372.7	2	186.4	F (2, 198) = 13.15	P<0.0001
Frequency oscillations	1794	5	358.7	F (5, 198) = 25.31	P<0.0001
Residual	2806	198	14.17		

S4 Table. MRCP correlation for the tasks relax, alert and grasped movement for different pair of electrodes

Electrode combinations	Relax	Alert	Movement
P4-P3	0.812510844005758	0.882816810591433	0.795689280510552
P4-C4	-0.017961502683891	0.810722568934	0.822053383861466
P4-F4	0.690504458848669	0.520982372276546	0.676572512552146
P4-C3	0.575374981540594	0.781414550296851	0.840684114364046
P4-F3	0.604688066224822	0.451575273564733	0.641578741996817
P3-C4	-0.0792461658319092	0.751878344550736	0.681687848253903
P3-F4	0.63335699227134	0.366363932435249	0.659984977723253
P3-C3	0.5750691218989	0.70909751525645	0.695708591467635
P3-F3	0.520549747388169	0.44419621495313	0.738511651398525
C4-F4	-0.0776127696858743	0.477144035686278	0.479651096467685
C4-C3	0.269929519451023	0.975872516821934	0.987481749736308
C4-F3	0.143989008452633	0.092062650136094	0.392546153308949
F4-C3	0.38875452061289	0.504870077458588	0.519706690242425
F4-F3	0.72606668074613	0.604054086090848	0.791482519155235
C3-F3	0.317659434357267	0.0631805823365063	0.405178959526388

Supervised learning

S5 Table. Model progression report for premovement and movement tasks for 14 electrode channels

Algorithm	Premovement_Movement		Left Movement & Right Movement	
	**Training Accuracy**	**Test Accuracy**	**Training Accuracy**	**Test Accuracy**
**LR**	62	47	50	48
**LDA**	52	47	56	43
**KNN**	50	47	51	56
**CART**	55	51	51	55
**NB**	52	51	50	46
**SVM**	57	52	49	48
**SVM_poly_3**	60	54	40	47
**SVM_poly_5**	60	52	53	56
**SVM_poly_7**	57	53	45	55
**SVM_poly_10**	55	55	45	55
**SVM_poly_13**	54	54	46	53
**SVM_poly_15**	53	54	46	52
**SVM_RBF**	57	52	49	48
**MLP (35,2)**	48	52	50	46
**MLP (35,5)**	56	50	4	42
**MLP (35,7)**	55	52	52	48
**MLP (35,10)**	60	44	55	50
**MLP (35,2,300,)**	50	52	50	46
**MLP(35,2,500,)**	50	52	50	46
**MLP(35,5,300,)**	62	47	50	45
**MLP(35,5,500,)**	62	47	50	45
**MLP(65,2,300,)**	50	52	50	46
**MLP(65,2,500,)**	50	52	50	46
**MLP(65,5,300,)**	54	49	46	45
**MLP(65,5,500)**	54	49	46	45
**DT**	54	50	53	45

S6 Table. Machine learning based model performance among the central electrode regions for the tasks premovement and grasped movement, left direction grasped movement and right direction grasped movement. The Accuracy rate table including the algorithms DT, SVM (polynomial Kernel, degree 3,5,6,10,13) SVM (radial basis function), MLP (No of Layers,2,5,7,10)

Algorithim	Premovement_Movement		Left Movement_Right Movement	
	**Training**	**Testing**	**Training**	**Testing**
DT	65	33.33333	67.5	66.66667
SVM	64.16667	50	73.33333	83.33333
SVM_poly(`3)	70	50	76.66667	83.33333
SVM_poly(`5)	66.66667	50	76.66667	83.33333
SVM_poly(`7)	66.66667	50	76.66667	83.33333
SVM_poly(`10)	66.66667	50	76.66667	83.33333
SVM_poly(`13)	66.66667	50	76.66667	83.33333
SVM_rbf_C2(`3)	64.16667	50	73.33333	83.33333
MLP2	64.16667	50	53.33333	100
MLP5	63.33333	33.33333	55.83333	100
MLP7	60	16.66667	55.83333	100
MLP10	60	16.66667	55	100

S7 Table. Accuracy scores and F1 test parametric for the central electrode regions.

	Premovement_ Movement			Left Movement _ Right Movement		
Algorithm	Accuracy (test)	F1score	AUC	Accuracy (test)	F1score	AUC
DT	33.33	0.33	0.33	66.67	0.66	0.66
SVM	50	0.5	0.66	83.33	0.833	1.0
SVM (Poly ‘3)	50	0.5	0.55	83.33	0.833	1.0
SVM (RBF)	50	0.5	0.3	83.33	0.833	1.0
MLP	50	0.5	0.5	100	1.0	1.0
